# Timing of dietary effects on the epigenome and their potential protective effects against toxins

**DOI:** 10.1080/15592294.2025.2451495

**Published:** 2025-01-18

**Authors:** Lynnea A. Nicholls, Kendall A. Zeile, London D. Scotto, Rebecca J. Ryznar

**Affiliations:** a Rocky Vista University College of Osteopathic Medicine, Parker, CO, USA; b Department of Biomedical Sciences, Rocky Vista University College of Osteopathic Medicine, Parker, CO, USA

**Keywords:** Epigenetic modifications, environmental toxins, transgenerational effects, epigenetic duration, DNA methylation, HDAC, miRNA, dietary protection

## Abstract

Exposure to toxins causes lasting damaging effects on the body. Numerous studies in humans and animals suggest that diet has the potential to modify the epigenome and these modifications can be inherited transgenerationally, but few studies investigate how diet can protect against negative effects of toxins. Potential evidence in the primary literature supports that caloric restriction, high-fat diets, high protein-to-carbohydrate ratios, and dietary supplementation protect against environmental toxins and strengthen these effects on their offspring’s epigenome. Most notably, the timing when dietary interventions are given – during a parent’s early development, pregnancy, and/or lifetime – result in similar transgenerational epigenetic durations. This implies the existence of multiple opportunities to strategically fortify the epigenome. This narrative review explores how to best utilize dietary modifications to modify the epigenome to protect future generations against negative health effects of persistent environmental toxins. Furthermore, by suggesting an ideal diet with specific micronutrients, macronutrients, and food groups, epigenetics can play a key role in the field of preventive medicine. Based on these findings, longitudinal research should be conducted to determine if a high protein, high-fat, and low-carbohydrate diet during a mother’s puberty or pregnancy can epigenetically protect against alcohol, tobacco smoke, and air pollution across multiple generations.

## Toxin burden on public health

In the United States, pervasive environmental toxins such as air pollution, tobacco smoke, and alcohol present daily challenges to public health. Notably, air pollution constitutes a significant concern, affecting nine out of ten individuals in urban areas [1]. Approximately 11.5% of US adults engage in cigarette smoking [2], while alcohol consumption is prevalent among 84% of the adult population [3,4]. The vast majority of US residents encounter these toxins at various points in their lives. These environmental exposures have been linked to epigenetic modifications [5–12].

However, the precise implications of these epigenetic modifications, as well as the process of learning how to protect against them through the utilization of preventive medicine, remain insufficiently understood [13].

To reiterate, epigenetic modifications represent heritable changes without altering the underlying DNA sequences, the effects of which include changes to chromatin structure and gene expression [14]. These epigenetic modifications occur in the individual who encounters the toxin and can then be inherited by their offspring, whether or not they are also exposed to the same toxin. These inherited effects, and how long they endure throughout generations, are the epigenetic effects that will be highlighted in this literature review with respect to dietary intervention, duration of change, and timing. As a result of this, this literature review will highlight aspects of inherited epigenetic changes in hopes of being a novel addition to the rapidly expanding academic discussion on epigenetics. Several studies provide evidence supporting the transgenerational inheritance of epigenetic modifications across varying species [11,15–23].

This literature review focuses on the interaction between environmental toxins and the epigenome, with proposed intentional dietary regimens that may protect individuals from the harmful effects of such toxins. Based on the evidence found in the literature, the goals of this paper are to discuss three key points: (1) how the timing of a dietary intervention can influence the duration of its epigenetic effects, (2) how priming the epigenome can increase its plasticity, and (3) how knowledge in these areas can help us create an ideal prescription diet. This diet would include specific micronutrients and macronutrients designed to protect future generations against toxin exposure.

Some terms that are integral to understanding this review include epigenetic plasticity, epigenetic priming, transgenerational epigenetic duration, and oxidative stress. Epigenetic plasticity refers to when the epigenome is particularly susceptible to epigenetic modifications [15]. Plasticity occurs during periods of rapid growth and maturation, such as during embryogenesis, early development, puberty, and pregnancy [24,25]. Epigenetic priming is when the chromatin structure is altered through processes such as histone modification or DNA methylation, and this enhances or diminishes a cell’s sensitivity to external signals without changing the underlying DNA sequence [14]. Transgenerational epigenetic duration is defined in this paper as the number of generations that an epigenetic change remains in the genome for. Finally, oxidative stress is an accumulation of reactive oxygen species within the body that exceeds the body’s detoxification pathways and leads to dysfunction in cellular metabolism, cell death, and systemic inflammation [26–31].

## Oxidative stress and disease states

Reactive oxygen species, which are produced during various metabolic processes and as a result of environmental toxins, can cause significant DNA damage [26–29]. Accumulation of reactive oxygen species within cells leads to oxidative stress, which contributes to systemic inflammation and diseases such as cardiovascular disease, chronic obstructive pulmonary disease, neurodegenerative disorders, chronic kidney disease, diabetes, sarcopenia, and cancer [27]. However, the harmful effects of oxidative stress can be reduced through nutrition, specifically by consuming natural antioxidants like vitamin E, vitamin C, polyphenols, and carotenoids found in common foods [30].

Our bodies accumulate oxidative stress from both internal (endogenous) and external (exogenous) sources. Endogenous sources include enzymes like NADPH oxidase, myeloperoxidase, and lipoxygenase, which are produced during normal physiological processes, such as cell metabolism, immune defense, and receptor or ion channel activation for cell signaling [32]. Exogenous sources of toxins include pollutants in air and water, tobacco, alcohol, heavy metals, drugs, industrial solvents, processed seed oils, smoked or charred meats, and radiation. While some of these toxins, like alcohol and charred meats, are consumed voluntarily, others, such as air pollution [33], secondhand smoke, heavy metals and pesticides, are encountered involuntarily. An imbalance between these toxins and the body’s detoxification systems or antioxidants can lead to cellular dysfunction, disrupted metabolism, and even cell death [30,31].

Antioxidants mitigate oxidative stress by donating electrons to harmful free radicals, neutralizing their ability to cause cellular damage [30]. Additionally, toxins are metabolized in the body through the Cytochrome P450 system, specifically by the *CYP1A1* and *CYP1A2* genes. *CYP1A1*, which is abundant in the kidneys, liver, and intestines, plays a crucial role in Phase I metabolism in the liver. This process involves oxidation, reduction, and hydrolysis reactions [34], all of which make molecules more polar and water-soluble, facilitating their excretion from the body.

As discussed above, oxidative stress is a primary mechanism through which toxins alter the epigenome. This can lead to downstream effects such as DNA methylation, histone modifications, and chromatin remodeling, which collectively contribute to cellular dysfunction, damage, or death, ultimately resulting in changes to the epigenome.

## Defining epigenetics and epigenetic modifications on the epigenome

In addition to the damaging effects of oxidative stress on DNA, epigenetic modifications also play a crucial role in regulating DNA expression. These modifications are marks above the DNA sequence that are passed down from generation to generation, impacting the way genes are transcribed and translated. Some of these modifications can influence disease pathogenesis, genomic imprinting, and mediate effects of the environment on DNA methylation and other covalent modifications, including histone remodeling and RNA modification [35–37].

### DNA methylation and histone modification

DNA methylation is typically measured with cytosine residues in CpG islands and utilizes enzymes such as DNA methyltransferase (DNMT) [36]. Histone modification includes methylation, acetylation, phosphorylation, ubiquitination, among others [14,38]. One way histone modification can be measured is by relative density of histone deacetylase (HDAC), which removes acetyl functional groups from histone proteins and increases transcription availability for the genes associated with those histone modifiers. Specific epigenetic modifiers of RNA include microRNA (miRNA) [37], transferRNA (tRNA) [39], and lncRNA [40,41] which function by reducing the variability of expression of genes with which they are associated. Moreover, covalent modifications to the epigenome via DNA, RNA, and histones, influenced by dietary nutrients, show promise in underlining the interplay between cellular mechanisms and environmental factors in shaping gene expression.

Covalent modifications to the epigenome via DNA, RNA, and histones can all be influenced by dietary nutrients and their associated covalent modifications to DNA [5–12]. Nutrients from our diet can upregulate or downregulate the expression of enzymes involved in these epigenetic processes and, in many cases, increase methylation of genes whose expression is unwanted as a result of increased inflammation. For example, when genes encoding for HDAC are methylated by the addition of the micronutrient resveratrol, this can lead to decreased expression of pro-inflammatory markers such as TNF-alpha [42,43].

Methylation, or one-carbon metabolism, is a series of metabolic pathways in which one-carbon groups are transferred from donors and used to methylate other reactions. This pathway is crucial for cellular function as well as the synthesis of DNA, amino acids, and phospholipids [44]. For example, folate, choline, and other B vitamins act as methyl donors and cofactors for the synthesis of a cosubstrate S-Adenosyl methionine (SAM), which stimulates DNA methylation reactions [45]. Furthermore, while epigenetic modifications introduce dynamic alterations to gene expression, genetic modifications remain static and are typically conserved, subject only to alterations through mutations.

### Dynamic nature of epigenetic modifications

Epigenetic modifications act as dynamic alterations to gene expression that increase an organism’s adaptability to the environment. Over time, gene mutations lead to genetic drift, resulting in permanent changes to a species’ population, which enable it to evolve and become more adapted to its environment [46]. Sometimes, however, there may be alterations to the environment that change faster than genetic drift, making some genes inconsequential. In these instances, silencing genes that are no longer needed or expressing ancient genes that are once again relevant, can be faster and more efficient for an organism. This is accomplished through epigenetic modification. As environments undergo gradual transformations across generations, corresponding epigenetic modifications diminish. These alterations occur to facilitate new and adaptive gene expression suitable for the dynamic environmental surroundings [35]. Of these modifications, dietary nutrients play an integral role in determining the number, nature, and extent of epigenetic modifications across an individual epigenome.

## Epigenetics and diet

Epigenetics can be positively influenced by diet to increase adaptability [8,12,42,47] or negatively influenced by diet in a way that decreases adaptability [22]. Conversely, epigenetics can also be negatively altered by environmental toxins [5–12,47]. If the negative effects that some environmental toxins have on genes can be linked to the positive effects that some dietary interventions have on genes, the diet may have a protective effect against epigenetic harm done by toxins [48]. For example, in Shen et al, a key comparison is made between protein to carbohydrate ratios and involvement in the MAPK pathway. This pathway is involved in many different cellular functions such as proliferation, apoptosis, differentiation, metabolism, and inflammation [49]. Cigarette smoke can also induce changes in this pathway that lead to pulmonary inflammation [48]. Alternatively, a high protein to carbohydrate diet can have an adaptive effect on the MAPK signaling pathway, increasing longevity and fecundity and decreasing inflammation [16]. This is just one of many examples of how diet can have similar, but antagonistic epigenetic interactions brought on by toxin exposure [32]. Once connections like these are found, moments of greatest epigenetic plasticity and nutrients with the greatest epigenetic effect can then be determined to propose an ideal diet that provides a maximum benefit to offspring.

## Epigenetic plasticity and the modern environment

Similarly to the previously mentioned epigenetic modifications through toxins, oxidative stress, and diet, epigenetic plasticity can play an integral role in transgenerational expression. As described above, epigenetic plasticity is when the epigenome is particularly susceptible to the mentioned modifications [15] such as during embryogenesis, early development, puberty, and pregnancy [24,25]. During these periods of rapid growth, genes such as *Dppa2* and *Dppa4* (developmental pluripotency-associated) are expressed and actively remodel chromatin to maintain opportunities for epigenetic reprogramming and differentiation [25,50]. *Dppa2* and *Dppa4*, for example, remain demethylated until 7.5 d after birth of an embryo to allow for proper differentiation and gamete formation [50]. Recognizing that certain time periods in the lifespan render an organism more susceptible to epigenetic modifications and acknowledging the existence of macro and micronutrients capable of targeting specific sequences for methylation or acetylation alterations, it becomes paramount to determine how to integrate this information.

## Transgenerational duration across species & diet intervention

When looking at the duration of epigenetic change after implementation of a dietary intervention in various animal species, evidence has shown that the timing of intervention and the sex of the parent given the intervention do not significantly impact transgenerational duration of epigenetic change. Priming the epigenome and accumulation of change with persistent intervention, however, do result in significant change in duration.

When surveying studies for use in collecting transgenerational epigenetic modification data, the studies had to have epigenetic modification data collected from the genome of at least two subsequent generations. Keywords such as ‘diet,’ ‘pregnancy,’ ‘epigenetics,’ and ‘transgenerational effects’ were entered into PubMed, Google Scholar, and EBSCO.

The selection criteria for the studies included in Tables 1 and 2 were selected based on their methodological quality, relevance to the research focus on dietary interventions and epigenetic modifications, and their investigation of transgenerational effects. The scope included diverse animal and human models, a range of dietary interventions such as high-protein diets, caloric restriction, and supplementation with specific micronutrients, and varying timelines of dietary administration (pre-fertilization, pregnancy, or across the lifespan). Studies older than 10 y were excluded to ensure the inclusion of recent advancements in the field.

**Table 1.** Description of the 10 articles that investigate various diets, their epigenetic effect, and their transgenerational duration in variable species.

Species	Dietary Intervention	Sex & Timing of Intervention	Generations Involved	Effects Measured
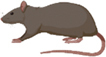	70% calorie restriction	Maternal pregnancy & lactation	F0 fed diet; F1 given SD or HFD; F2 studied	Lipogenesis gene expression
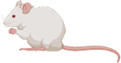	Resveratrol & high fat	Maternal prefertilization	F0 fed diet; F1-F2 studied	Dnmt1 & Dnmt3a gene expression
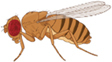	Protein:carbohydrate ratios	Maternal & paternal prefertilization	F0 fed diet; F1-F3 studied	Longevity & fecundity
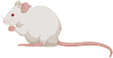	Vitamin D deficiency	Maternal prefertilization	F0 fed diet; F1-F2 studied	H19/IGF2, Snrpn, Dlk1/Glt2, Grb10 methylation
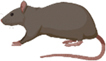	Arachidonic acid supplementation	Maternal pregnancy & lactation or Paternal prefertilization	F0-F2 fed diet; F1-F3 studied	Scd1 promoter methylation
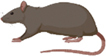	50% calorie restriction	Maternal pregnancy	F0 fed diet; F1-F3 studied	Dnmt1, Dnmt3a, Dnmt3b, Mecp2, Hdac1, Sin3a transcription
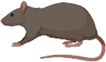	High fat	Maternal pregnancy	F0 fed diet; F2 studied	Dlk1 imprinting
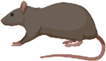	50% calorie restriction	Maternal pregnancy	F0 fed diet; F1-F3 studied	Pld1 & Oxct2b transcription
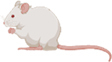	High fat	Maternal lifespan	F0 fed diet; F2-F3 studied	STAT3, STAT5, NeuroD1 methylation
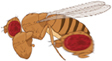	Protein:carbohydrate ratios	Maternal & paternal lifespan	F0-F1 fed diet; F3 studied	MAPK pathway & ribosome biogenesis gene transcription

**Table 2.** Summarization of protection articles with gene, dietary intervention, toxin, and how the gene was modified by the dietary intervention.

Species	Gene Target	Dietary Intervention	Toxin	Effects Measured
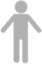	AHRR	Mediterranean Diet	Tobacco smoke	Increased methylation of AHRR
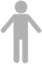	AHRR	Provitamin A	Tobacco smoke and ethanol	Increased methylation of AHRR
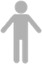	AHRR	Folic Acid	Tobacco smoke	Increased methylation of AHRR
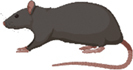	HDAC	Resveratrol	Morphine	Decreased HDAC expression
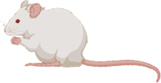	HDAC	EGCG, tea polyphenol	Hematopoetic radiation injury	Decreased HDAC expression
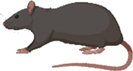	miRNA	Choline	Ethanol	Decreased induction of miRNA
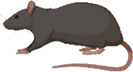	miRNA	Folic Acid	Persistent Organic Pollutants	Decreased disruption of miRNA
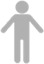	CPO, DAXX, GLI2	B vitamins	Ambient fine particles	Decreased methylation of DNA

The interventions evaluated covered a broad spectrum of strategies, including macronutrient manipulation, micronutrient supplementation (e.g., folate, resveratrol), and their effects on epigenetic markers such as DNA methylation, histone modification, and miRNA expression. These studies also explored the interaction between diet and toxin exposure, focusing on mechanisms that might mitigate the epigenetic damage caused by substances like tobacco smoke and ethanol.

Synthesizing these findings presented challenges due to the heterogeneity of species studied, dietary protocols, and outcome measures. Variability in data collection methods and confounding environmental factors, such as psychological stress or additional exposures, also complicated comparisons. The specific limitations from each study method analyzed are listed in Table 3. Despite these limitations, the studies provide robust evidence supporting the protective role of dietary interventions against toxin-induced epigenetic changes and their transgenerational persistence.

**Table 3.** More detailed description of the 18 articles that investigate various diets, their epigenetic effect, their transgenerational duration, and their potential to protect against environmental toxin exposure.

	Species	Dietary Intervention	Timing of Intervention	Generations Involved	Toxin Exposure	Effects Measured	Limitations
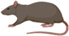		RD = 70% FR30, HFD = 45.8% fat, SD = 8.4% fat	Given to F0 mother during pregnancy & lactation	F0 fed diet; F1 given SD or HFD; F2 studied	None	Lipogenesis gene expression	Study discontinued at F2, did not look at later generations
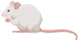		1 g/kg resveratrol (RSV) within diet of 16:23:61 carb/protein/fat ratio	Given to F0 mother from 21 d old to 3 months old, prior to fertilization	F0 fed diet; F1-F3 studied	None	Dnmt1 & Dnmt3a gene expression	
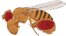		LP = 3.3% protein 90.5% carb, IP = 5.5% protein 87.5% carb, HP = 13.5% protein 69.5% carb	Given to F0-F2 mother during pregnancy & lactation	F0-F2 fed diet; F1-F3 studied	None	Longevity & fecundity	Did not distinguish male vs female contributions
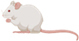		SD = 1000 IU/kg vitamin D3, Vit D deficient = 0 IU/kg vitamin D3	Given to F0 mother 5 weeks before mating & through weaning	F0 fed diet; F2-F3 studied	None	H19/IGF2, Snrpn, Dlk1/Glt2, Grb10 methylation	True evidence of subtle epigenetic change undetermined
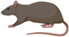		98.5% pure arachidonic acid within vehicle oil SD=vehicle oil only	Given to F0-F2 mother during pregnancy & lactation	F0-F2 fed diet; F1-F3 studied	None	Scd1 promoter methylation	Effects of AA metabolism vs FA activity cannot be distinguished
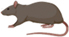		RD = 50% of typical SD AIN-93 G diet ad libitum	Given to F0 mother for 19 d of pregnancy	F0 fed diet; F1-F3 studied	None	Dnmt1, Dnmt3a, Dnmt3b, Mecp2, Hdac1, Sin3a transcription	Genome wide analysis not completed
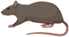		HFD = 45% fat58V8 TestDiet SD = 5755 TestDiet	Given to F0 mother during pregnancy	F0 fed diet; F1-F3 studied	None	Dlk1 imprinting	Study discontinued at F2, did not look at later generations
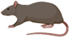		RD = 50% of typical SD AIN-93 G diet ad libitum	Given to F0 mother for 19 d of pregnancy	F0 fed diet; F2-F3 studied	None	Pld1 & Oxct2b transcription	Looked at transcripts and not protein itself
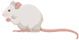		HFD = 23% protein 42% carb 60% fat, SD = 18.5% protein 46% carb 6.55% fat	Given to F0 mother from 1 month of age to second week of lactation	F0 fed diet; F1-F2 studied	None	STAT3, STAT5, NeuroD1 methylation	
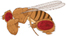		Protein to Carbohydrate ratios of 1:2, 1:4, 1:6	Given to F0 mother & father during their whole life	F0-F1 fed diet; F3 studied	None	MAPK pathway & ribosome biogenesis gene transcription	Confounding data due to known metabolic effects of white Drosophila gene
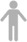		Adherence to Med Diet (0–17 points) 08=low 9–17=high	N/A	One generation	Self-reported tobacco smoking former, never, or current	Increased methylation of AHRR	Difficult to compare sex differences due to X inactivation
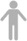		Measured serum levels of provitamin A: 0.153–0.990 umol/L	N/A	One generation	Self-reported tobacco smoking & drinking former, never, current	Decreased HDAC expression	Cross sectional study, should do longitudinal
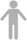		Maternal serum folate level of >19.2 nmol/L	N/A	One generation	Positive self-reported tobacco smoking during pregnancy	Decreased HDAC expression	Low birthweight and preterm babies self-reported smoking habits
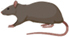		Subsequent intrathecal injection of 30 mcg resveratrol	N/A	One generation	1.5 hour post-irradiation to lethally whole body irradiated mice	Decreased induction of miRNA	
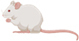		0.1833 mg/kg EGCG	N/A	One generation	15 mcg/hr of morphine or saline for 120 hours	Decreased induction of miRNA	
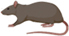		100 mg/kg/day choline chloride or saline from postnatal day 4–21	N/A	One generation	5.25 g/kg/day ethanol from postnatal day 4–9	Decreased induction of miRNA	miRNA only examined during one developmental timepoint
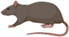		2 mg/kg folic acid or 6 mg/kg folic acid	N/A	One generation	500 mcg polychlorinated bisphenyls (PCBs)/kg 3× per week for 9 weeks	Decreased induction of miRNA	
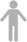		2.5 mg/day folic acid, 50 mg/day vitamin B6, and 1 mg/day vitamin B12	N/A	One generation	2 hour exposure to PM2.5 (250 mcg/m^3)	Decreased induction of miRNA	n = 10; could not randomize order of treatment; findings might not be generalized to other cell types

RD = restricted diet, SD = standard diet, HFD = high fat diet, LP = low protein, IP = intermediate protein, HP = high protein.

### Epigenetic duration

**Figure 1.**
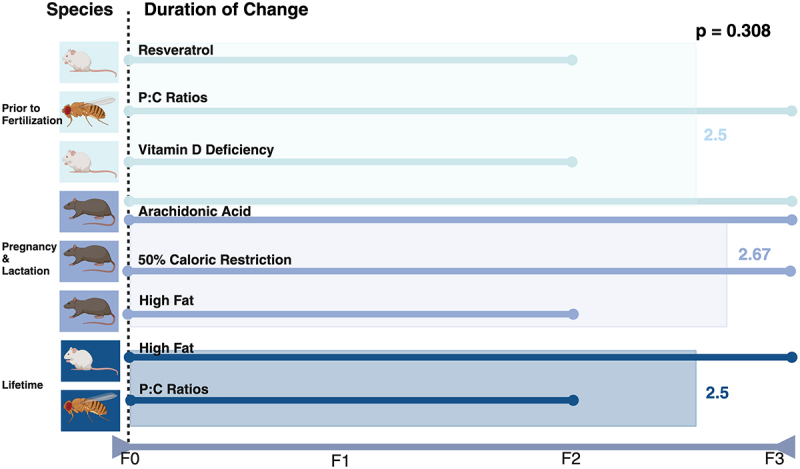
The duration of epigenetic change is graphed on the y-axis with the average duration for each timing of dietary intervention graphed in transparent blocks. This figure uses data from the 10 articles outlined in table 1.

It has been shown that epigenetic modifications on a fetus and infant from the mother’s diet during pregnancy and lactation can be passed down to future offspring [51]. As evidenced by the data from animal studies in Table 1, however, the particular moment in the mother’s lifespan when the dietary intervention is introduced does not significantly alter the duration of epigenetic change. A diet can be consumed by the F0 generation prior to fertilization, during pregnancy, pregnancy and lactation, or across the lifespan with a similar effect on duration of epigenetic change. These epigenetic changes can be passed down for two to three generations, regardless of when the F0 parent consumes the epigenetically influential diet (See Figure 1).

While dietary interventions during early development and pregnancy are widely recognized as critical periods for influencing offspring epigenetics, less is understood about how lifetime dietary habits of parents contribute to transgenerational epigenetic inheritance. Evidence suggests that consistent dietary exposures throughout a parent’s life may gradually reshape the epigenetic landscape of germ cells via mechanisms such as cumulative DNA methylation changes, histone modifications, and non-coding RNA regulation. These changes can persist across generations, emphasizing the need for further research into the molecular pathways that underpin such long-term effects. Understanding these processes will provide insight into leveraging lifetime dietary behaviors as a preventive measure against transgenerational health risks.

This provides more opportunities for the epigenome to be beneficially altered by diet as well as harmfully altered by toxins and subsequently passed onto offspring. While human exposure to many toxins cannot be controlled, the diet can be used as a defensive line against these uncontrollable harms [52]. Furthermore, diet may be consumed during pre-fertilization, pregnancy, lactation, or the entire lifespan to induce a protective impact on the epigenome that could be passed down for two to three generations (See Figure 1). Importantly, this dual potential of diet to either protect or harm emphasizes the need for increased awareness of dietary choices and their long-term implications. As such, the ability to mitigate environmental harm through controlled dietary practices highlights diet as a modifiable factor with transgenerational impact, underscoring its critical role in shaping the health of future generations. Future research should focus on elucidating the precise molecular mechanisms driving these epigenetic changes, as well as the optimal timing and composition of dietary interventions to maximize transgenerational health benefits while minimizing risks. This presents an opportunity to advance our understanding of diet as a tool for epigenetic modulation and public health improvement.

### Maternal versus paternal impact

The literature suggests that sex of the parent given the dietary intervention does not have a significant impact on the duration of epigenetic change [16,18]. There is significant evidence from both human and animal studies that transgenerational epigenetic modifications can be passed down from both parental sexes rather than just the female sex, responsible for providing the in utero environment [53]. Transgenerational inheritance of longevity due to protein to carbohydrate ratio variability in drosophila remained ancestral sex-independent [16]. Additionally, *Scd1* promoter regulation, which has an important role in lipid metabolism, energy balance, and storage and oxidation of lipids [54], showed similar trends when the male, female, or both male and female rat parents were given arachidonic acid supplementation [18].

## Methods that suggest an increase in epigenetic transgenerational duration

There does not seem to be a significant difference in the duration of epigenetic change based on timing of dietary intervention in the F0 generation (see Figure 1). However, there may be alternative ways to increase significance or duration of epigenetic change passed to offspring. One way research has shown this is via epigenetic priming by way of nutrient deprivation [15,19,20].

Epigenetic priming, which involves altering chromatin structure through processes such as histone modification or DNA methylation, can enhance or diminish a cell’s sensitivity to external signals without changing the underlying DNA sequence [14]. This review aims to understand the relationship between priming, environmental factors, while highlighting diet modifications, as well as their potential to protect against environmental toxins and influence the epigenome for future generations.

### Thrifty hypothesis visited

When looking at the effects of nutrient deprivation across multiple generations, nutrient deprivation in F0 could act as an epigenetic priming mechanism to prepare the offspring’s epigenome to rapidly adapt to any future diet it is presented with, according to the literature [15,19,20].

Using 50% dietary restriction as the dietary intervention, one study looked at DNA methylation in hepatic cells of genes involved in metabolic processes and measured their methylation across three generations of rats, finding no significant results in the transgenerational epigenetic effects caused by dietary restriction in F0^(19)^. Another study looked at the methylation and expression of various genes in hepatic DNA, and found that *HDAC1* demonstrated increased expression and activity (less methylation) until the F3 generation [20]. While restricted diet during pregnancy may not alter hepatic genes involved in metabolic processes, it could induce changes in the expression of genes associated with regulation of epigenetic modifications, given that the expression and activity of *HDAC1* plays a prominent role in the ability to alter the epigenome at large [19,20]. This timeframe then primes the mechanisms of epigenetic change in the cell to make additional epigenetic changes in the presence of the variable diets of future generations.

While dietary restriction does impact the epigenome of genes involved in metabolic processes for the generation consuming the restricted diet, it does not pass these changes down to the next generation [19]. Dietary restriction does, however, alter the expression and activity of genes associated with regulating epigenetic modifications, for up to three generations [20]. This displays epigenetic priming, where the restricted diet in the F0 generation activated the epigenetic mechanisms to be ready to methylate, acetylate, and alter the expression of metabolic genes in future generations based on the diet they are presented with in their life, whether it be more restriction, abundance, high fat, or high protein, etc.

Epigenetic priming by first generation calorie restriction is further explored in Cissé et al. In this study, the F0 generation was given a 70% calorie restricted diet and the F1 generation was subsequently given a high fat diet. Insulin resistance, corticosterone production, and expression of lipoproteins were studied in F3. It was found that the F3 generation in the experimental group, where F0 was given a calorie restricted diet, had an improved metabolic health compared to F3 individuals whose F0 ancestor was not calorie restricted. This ancestral calorie restriction in one generation presented an opportunity for increased adaptability to varying diets in later generations [15]. Calorie restriction, however, is not always a beneficial epigenetic priming intervention [55].

Extreme caloric restriction has not been shown to be beneficial over the long term, as it leads to growth restriction in offspring and inability to reach full strength and size [15,55]. In the context of this study, however, caloric restriction in F0 allowed for increased metabolic health in F2. This displays how challenges in the environment, such as famine in an ancestral generation, can lead to increased adaptability of offspring when facing challenges of a similar nature. For example, the caloric restriction in F0 led to increased epigenetic plasticity of the hepatic cells in F1, making them more efficient in lipid metabolism. This study then follows the next generation, F2, to see how the diet of F1 alters the hepatic metabolism of F2. Regardless of whether F1 is on a control diet or a high fat diet, the liver of the F2 generation has greater metabolic health if their F0 ancestor was on a caloric restricted diet. More specifically, F2 has an augmented expression of lipid metabolization in the liver if their F0 ancestor was on a restricted diet. Conversely, F2 had an accumulation of fat in the liver of their F0 ancestor who was on the control diet. This study concludes that the calorie restricted diet of F0 primed the epigenome of F1 and F2 to have greater epigenetic plasticity, promoting greater liver metabolic adjustment to varying diets.

This finding parallels the ‘Thrifty Hypothesis.’ A proposed explanation of how nutrition deprivation in early life leads to an alteration in the glucose-insulin metabolism pathway, promoting increased fat storage. This is caused in part by a reduced capacity for insulin secretion and insulin resistance [56]. This response, while protective in preventing starvation in case of a future famine, actually may lead to metabolic disease and obesity in the context of caloric abundance [57–59]. So, how do we manipulate this epigenetic priming without causing the increased health challenges that the thrifty hypothesis displays in the current landscape? The answer may be in the percentage of caloric restriction and ensuring adequate micronutrient intake is maintained even in the context of caloric restriction [60–62]. More research should be done on how caloric restriction in one generation can positively impact the metabolic pathway of future generations. Additionally, research should be done on how epigenetic priming, similar to that caused by caloric restriction, can be accomplished in other cells of the body, in addition to hepatic cells.

### Multigenerational intervention accumulation

In addition to epigenetic priming through nutrient deprivation, research suggests another way to increase the protective epigenetic effect passed down to offspring may be by continuing a specific diet across multiple generations [18].

Looking at the cumulative effects of diet on the epigenome, one study using rat models demonstrated that arachidonic acid supplementation showed a cumulative effect on *Scd1* promoter demethylation, with demethylation being proportional to the mg of arachidonic acid given [18]. The *Scd1* gene has an important role in lipid metabolism, energy balance, and storage and oxidation of lipids [54]. Arachidonic acid is a precursor to numerous lipid mediators [63,64]. As arachidonic acid is repeatedly presented to the organism, there is likely evidence to suggest an increased need for *Scd1* in its metabolism, leading to progressive demethylation of the gene for increased expression and activity of *Scd1*.

In another study, the time to drosophila pupation remained the same if two generations or three generations were given a high protein to carbohydrate diet. The time to pupation is directly related to MAPK and ribosome biogenesis [23]. Sufficient protein intake is required for adequate growth and development [65]. A potential rationale noted lack of a cumulative effect after multiple generations of high protein to carbohydrate ratio diets, is that if two generations are given adequate protein intake, it may reach a point of diminishing returns. The organism may be able to grow and develop to the max of their ability without a need for increased protein intake [66].

**Figure 2.**
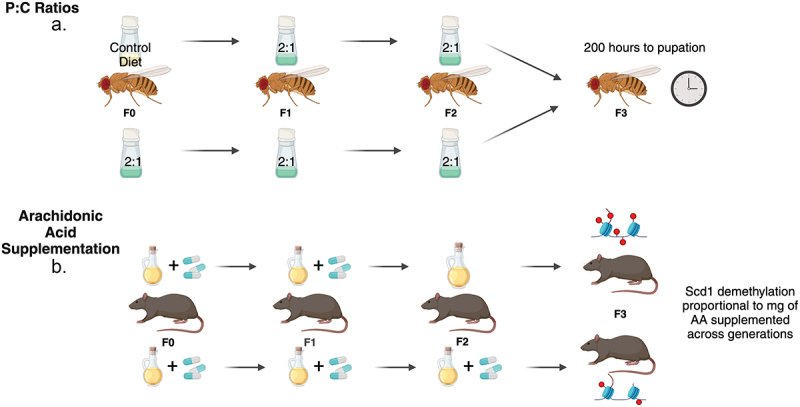
A. A 2:1 protein to carbohydrate ratio diet given for two generations has the same effect on time to pupation as the same diet given for three generations. It is not cumulative [23]. B. Amount of Scd1 demethylation is cumulative based on mg of arachidonic acid supplementation given across multiple generations [18].

Based on the above literature discussing duration of epigenetic change, beneficial epigenetic effects could be passed down for two to three generations even if F0 is the only one to consume the intervention. If there is a cumulative effect, however, it could be beneficial for generations F1 and F2 to also consume the intervention and pass on a greater beneficial impact to future generations (See Figure 2).

## Dietary intervention & toxin protection

As discussed in the literature, accumulating dietary interventions across multiple generations and priming the epigenome with a calorie deficit appears to have an impact on the duration of an epigenetic change passed on to future offspring while the timing of when the dietary intervention is given and the sex of the parent given the intervention does not appear to alter that duration. This information becomes pertinent when looking at implementation for protection of future generations against epigenetic insult from toxins. If we can decipher which dietary interventions affect the epigenome in a way that protects against epigenetic modifications from toxins, then consuming those diets can protect generations from toxins for as long the epigenetic change is passed down. For example, the research suggests Provitamin A, resveratrol, and choline provide significant improvement in the respective negative effects of tobacco smoke, morphine, and ethanol, typically measured in terms of DNA methylation, histone deacetylation, and miRNA expression.

Tobacco smoke contains numerous harmful chemicals, including but not limited to nicotine, formaldehyde, ammonia, carbon monoxide, benzopyrenes, tar, acetone, cadmium, and nitrogen oxides. These chemicals not only damage the epithelial cells throughout the respiratory tract but also cause harm at the level of DNA. Exposure to tobacco smoke is associated with changes in DNA methylation, which affects genes linked to cancer, Alzheimer’s disease, diabetes, and neural development [67]. Additionally, tobacco and nicotine exposure negatively impact systemic inflammation, thyroid function, and cardiovascular function by altering miRNA [67]. This exposure also disrupts macrophage activity, which interferes with apoptosis, leading to an increase in DNA mutations and proliferation of potentially damaged cells [68].

A specific gene of interest in this context is *AHRR*, which is involved in metabolizing xenobiotic particles via the cytochrome P450 system [69–71]. Tobacco smoke causes hypomethylation of *AHRR*, which blocks *AHR* (Aryl hydrocarbon receptor) function^(5, 6, 10)^. As a result, increased *AHRR* expression reduces *AHR* effectiveness, decreasing the body’s ability to clear harmful particles introduced by smoking.

However, studies suggest that certain interventions may counteract these effects. For example, Tsuboi et al. demonstrated that provitamin A plays a protective role by hypermethylating *AHRR*, enhancing the clearance of tobacco smoke particles and restoring *AHR* function [6]. Similarly, the Mediterranean diet has been shown to hypermethylate *AHRR* and restore *AHR* effectiveness in individuals exposed to tobacco smoke [5]. Furthermore, research by Xu et al. and Zhang et al. links maternal smoking during pregnancy to *AHRR* hypomethylation and low folate levels [10,72]. Zhang et al. suggests that adequate folic acid intake during pregnancy may help counteract *AHRR* hypomethylation. Therefore, dietary interventions like provitamin A, the Mediterranean diet, and folic acid may mitigate tobacco smoke’s harmful epigenetic effects by counteracting *AHRR* hypomethylation and restoring *AHR* function.

Ethanol has negative effects on the DNA via multiple mechanisms. For example, the literature shows that exposure to alcohol increases histone acetylation as a result of decreased HDACs and HATs (histone acetyltransferases) and leads to a buildup of toxic metabolites [73]. Toxic metabolites of ethanol include acetate and acetaldehyde. Acetate can affect chromatin structure making it more relaxed and provoke ethanol-related behavioral manifestations. Acetaldehyde damages mitochondria by inducing oxidative stress and results in genomic instability due to the decreased detoxification of the damaged mitochondria [74]. Furthermore, ethanol increases aberrant expression of miRNA, which is involved in transcription. This aberrant expression of miRNA is related to some cancers [75]. However, in Balaraman et al, choline supplementation decreased miRNA variance and allowed for more homogenous expression of miRNA in rats who were exposed to ethanol [7].

Morphine is a known neuroinflammatory mediator and has been shown to increase levels of the inflammatory cytokines TNF-a, IL-1B, and IL-6 mRNA in morphine-tolerant rats [42]. Inflammatory cytokines are necessary in times of acute illness or injury, but inappropriate or excessive activation is associated with chronic inflammation, which leads to systemic disease [76]. Morphine also increases expression of HDAC [43], which is responsible for promoting a more closed chromatin structure, decreasing transcription. Izquierdo et al found that when resveratrol was added to Morphine, HDAC expression in rats decreased and neuroinflammation overall also decreased, specifically the concentration of TNF-a (pg/mg) [11]. Other studies included the role of EGCG, a tea polyphenol, in protecting mice against hematopoietic radiation injury [12].

It is important to acknowledge that many other environmental toxins, such as pesticides and heavy metals, can also alter the epigenome. While a brief description of their effects is provided below, this list is not exhaustive. These toxins were included to illustrate the diversity of environmental influences on the epigenome, but evaluating protective effects against these specific toxins was not the primary focus of our review.

Pesticides also significantly impact epigenetic mechanisms, including DNA methylation, histone modifications, and miRNA expression. Exposure to pesticides like Dichlorodiphenyltrichloroethane (DDT) and arsenic alters DNA methylation patterns, leading to gene-specific hypermethylation (e.g., *p53, RASSF1A*) and global hypomethylation, which disrupt gene expression and contribute to carcinogenesis [77]. Histone modifications, such as hyperacetylation caused by paraquat and dieldrin, affect chromatin structure, promoting neurotoxicity and transcriptional changes [77]. Additionally, pesticides like dichlorvos and fipronil disrupt miRNA expression profiles, impacting cellular signaling and gene regulation [77]. These epigenetic alterations underline the potential of pesticides to induce long-lasting changes in gene expression and increase disease susceptibility.

Furthermore, heavy metals, including arsenic, cadmium, nickel, and chromium, induce significant epigenetic alterations that contribute to their carcinogenic effects. These metals disrupt DNA methylation, causing gene-specific hypermethylation (e.g., *p16INK4a, MGMT, and hMLH1*), leading to silencing of tumor suppressor and DNA repair genes, while global hypomethylation promotes genomic instability [78]. They also affect histone modifications, with arsenic and cadmium inducing marks like H3K9me3 and H3K4me3 that silence or poise genes for activation, and nickel and chromium increasing histone deacetylation and H3K9me2 to compact chromatin and repress gene expression (e.g., *MGMT*) [78]. Short term exposure leads to rapid expression of immediate stress response genes such as *HSP70* and *c-jun/c-fos* [78]. Alternatively, long term exposure leads to more stable, heritable epigenetic alterations in chromatin structure, which can predispose cells to lasting gene expression changes (e.g., *MT-3*) [78]. Although less studied, heavy metals may also indirectly alter miRNA expression through these epigenetic changes, further modulating gene regulation and increasing disease risk [78].

When surveying studies for use in collecting toxin protective epigenetic modification data, the studies had to include a dietary intervention and subsequent protection against toxins. Keywords such as ‘diet,’ ‘toxin,’ ‘epigenetics,’ and ‘protective effects’ were entered into PubMed, Google Scholar, and EBSCO. Studies older than 10 y were excluded. All species were accepted, as well as a variety of dietary interventions and toxin exposures; however, special note was taken for studies found with dietary interventions that paralleled those found in the transgenerational studies found in Table 1.

**Figure 3.**
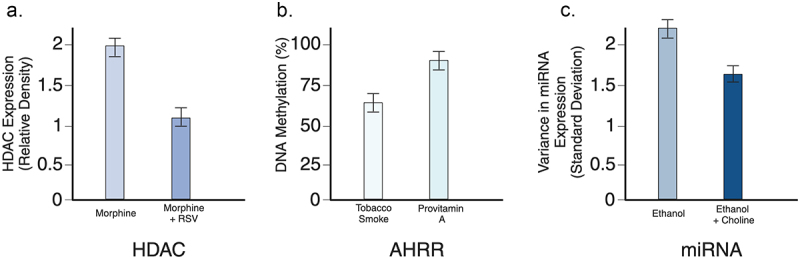
A. Compared relative density of HDAC expression for resveratrol vs morphine [40] (*p* value 0.005); B. Compared percent of DNA methylation between tobacco smoke vs provitamin a [6] (*p* value 0.034); C. Compared standard deviations in variance in miRNA expression for ethanol vs choline [7] (*p* value = 0.034).

See Table 2 and Figure 3 for summary of these studies. Additionally, please see Table 3 for a more detailed look at all of the studies included in the evaluation of epigenetic change duration as well as toxin protection. Further research is needed to explore additional protective agents against a variety of environmental toxins.

## Incorporating epigenetic protection through dietary intervention into a sustainable practice for patients

Epigenetic modifications through dietary intervention can be used as a means for providing more thorough and intentional patient care. By incorporating data on the duration of epigenetic effects resulting from dietary interventions, and coupling it with information regarding the protective effects of dietary interventions against various toxins, physicians can confidently initiate discussions on optimal dietary choices for leveraging our epigenome as a potent advocate for patient health.

The proposed methods for timing, priming, and accumulating dietary interventions can be overwhelming to an individual looking to bolster their epigenome against toxins. Furthermore, the myriad of ingredients that could protect against various toxins are plentiful and may be challenging to continually reference in a daily life. If we can combine the beneficial effects of the discussed foods into one comprehensive dietary recommendation, however, it may increase the likelihood of generations abiding by it and passing on benefits to their offspring. A notable diet that has been well-studied, the Mediterranean Diet, has been found to combine the beneficial effects of many of the dietary interventions discussed above [79], including high protein to carb ratios, inclusion of specific micronutrients and healthy fats.

Other dietary patterns, such as lower protein-to-carbohydrate ratios, low-fat diets, and the standard American diet, were not included as recommendations due to their detrimental effects on physiology, behavior, and recovery from injury, which are attributed to increased pro-inflammatory cytokines resulting from calorie-dense and nutrient-poor ingredients [80]. While other diets, such as ketogenic, paleo, and Atkins diets, demonstrate some short-term benefits – such as rapid weight loss and improvements in certain cardiometabolic markers – they were not included due to significant concerns regarding long-term adherence and potential negative health consequences.

The restrictive nature of these diets, particularly the extreme carbohydrate limitation in ketogenic and Atkins diets, often leads to high dropout rates in studies, suggesting challenges in sustainability for long-term health outcomes. Additionally, these diets have been associated with potential adverse effects, including unfavorable changes in lipid profiles (e.g., increased LDL-C), heightened risk of kidney stones and chronic kidney disease, and reduced bone mineral density over time. There is also evidence of metabolic acidosis and other complications, such as ketoacidosis, particularly in vulnerable populations like lactating women. Although some studies report initial improvements in metabolic and weight-related parameters, conflicting evidence about their long-term safety and effectiveness raises questions about their broader applicability as a universal recommendation [81].

### Components of the Mediterranean diet

The Mediterranean diet, one of the most studied diets in the literature, includes many of the dietary interventions mentioned previously: protein, healthy fats, and micronutrients such as folic acid, choline, Provitamin A, and resveratrol [82]. Because of this, it has the potential to act as a foundational diet that, when consumed consistently throughout the lifetime, can provide epigenetic protection against environmental toxins.

Protein in the Mediterranean Diet is sourced from whole grains, dairy products, fish, poultry, eggs, and red meat. Healthy sources of fat can be found in fish and other seafood, olives, nuts, and extra virgin olive oil. Finally, the micronutrients observed in the primary literature search can be found in abundance in the Mediterranean Diet. Folic acid, vitamin B9, is found in seafood and green leafy vegetables. Choline is found in eggs, red meat, legumes, and fish. Colorful fruits and vegetables are a major source of Provitamin A. Ascorbic acid, vitamin C, naturally occurs in many fruits and vegetables as well. Resveratrol, a polyphenol in the skin of grapes, can be found in wine, with a higher amount found in red wine [82,83].

Provitamin A, as discussed above, can improve clearance of harmful products in tobacco smoke via cytochrome P450. Folic acid can also modulate effects of tobacco smoke on AHRR [10]. Resveratrol decreases the neuroinflammation caused by morphine by downregulating HDAC and its activation of proinflammatory cytokines [11]. Choline helps to mitigate the aberrant expression of miRNA induced by ethanol, while folic acid helps to mitigate the aberrant expression of miRNA from persistent organic pollutants (POPs) [7,8]. Finally, B vitamins, in general, decrease the effects of ambient fine particles/air pollution on the genes *CPO*, *DAXX*, and *GLI2*^(47)^.

As evidenced by the above, there are multiple sources of these micronutrients found in the Mediterranean Diet. Additionally, focusing on whole foods and specifically a diversity of plant foods, would provide an individual with the nutrients necessary to best protect their epigenome.

### High fat diet versus Mediterranean diet

Some of the primary literature shows that a HFD was detrimental to the organism’s epigenome due to chromatin repression and subsequently alterations in hippocampal neural stem regulation [22]; However, it is important to note that the source and quality of fat should be taken into consideration. A typical HFD diet in animal research is typically a rodent diet with 60% kcal from fats [84]. When the source of the fat was investigated, this fat came from either lard or soybean oil. Research on soybean oil has proven to be inflammatory to the organism [85]. On the contrary, healthy fats from the Mediterranean diet, such as those in seafood and extra virgin olive oil, have been shown to decrease proinflammatory markers such as thromboxanes and leukotrienes [86] and decrease oxidative stress by down regulating oxidation of LDL and peroxidation of fatty acids [82]. The Mediterranean diet is considered to have a ‘moderate amount of fat’ sourced from high quality ingredients and has an abundance of data referencing overall decreased morbidity, mortality, metabolic syndrome, and cardiovascular disease [83], all of which inflammation is the underlying pathophysiology [82].

With regard to human trials, the literature is full of examples of detrimental effects of HFD ranging from gastrointestinal disease [87] to compromised female fertility [88], along with a myriad of other diseases. Once again, it is critical to distinguish these diets in the different ways they source fat. The high fat diet in these studies is likely related to highly processed foods that lack protein, fiber, vitamins, and minerals. The Mediterranean diet encompasses protein, healthy sourced fat, and other micronutrients that make it well-balanced, which is different from the HFDs references in the above trials.

### Use of the Mediterranean diet in the literature

In the literature, there are many differences in the classification of the Mediterranean diet. Per Mayo Clinic, the general definition of the Mediterranean Diet is as follows: emphasis on variety of plant foods including vegetables and fruit, nuts, and seeds; whole, unrefined, fiber rich foods and grains such as beans, legumes, brown rice; plenty of extra virgin olive oil (EVOO) and fish as a healthy source of omega-3 fatty acids; low to moderate amount of cheese and yogurt; little to no meat, choosing poultry over red meat; little to no sweets, sugary drinks, or butter; and moderate amount of wine with meals, only if the patient already drinks wine.

Most of the discrepancies seen in the Mediterranean Diet definition focus on different percentages of macronutrients. For example, fat can make up between 25 and 40%, carbohydrates ranging from 40 to 60%, and protein between 15 and 35% [89,90]. The adaptable nature of this diet means there is no strict formula. This provides flexibility and heterogeneity, making it sustainable and realistic for the average individual, while also complicating the process of conducting research because of the variable dietary instructions. This does pose a challenge, but multiple studies have been conducted to test the sustainability and adaptability of the Mediterranean diet for various cultures and income levels. For example, the Trial to Encourage Adoption and Maintenance of Mediterranean Diet (TEAM-MD) looked at how the MD could be adapted to account for more common locally sourced foods. They determined that while this made measuring its specific health benefits challenging due to the diet’s heterogeneity, it was important to learn how to continue those studies with a variable diet in order to allow for adaption of the diet to various cultures and geographic locations [91]. In another example, Estrada Del Campo et al looked at the sustainability of the MD for low-income Chilean women at risk for cardiovascular disease. In this case, the adapted MD allowed for these women to report high engagement and acceptability, as well as improved self-perceived dietary habits [92,93].

While there have been many longitudinal human trials utilizing the Mediterranean diet as an intervention in patients with cardiovascular disease and/or metabolic syndrome [91–95], these studies have not specifically looked at the transgenerational epigenetic changes caused by eating the Mediterranean diet during various periods in the lifespan.

With the Mediterranean diet as a recommended dietary foundation, there are innumerable opportunities to see protective epigenetic effects. Furthermore, with the Mediterranean diet being adapted to become accessible and sustainable to various populations, the protective epigenetic effects can also be made more accessible.

## Limitations to application

While there are no human species utilized in the discussion on duration of epigenetic change, this is due to the complexities of following multiple generations of human subjects. Some limitations in interpreting the information from this review, therefore, are the lack of longitudinal human trials. The presence of longitudinal human trials and their relation to this analysis is included in the conversation on epigenetics and toxin exposure; however, human trials as it relates to toxin exposure and protective dietary intervention is limited due to ethical concerns. Therefore, the research has not been significantly verified that these changes will result in transgenerational benefit. Transgenerational examples from the primary research can be found in Table 2 with *AHRR* comparing folic acid and tobacco smoke [10] as well as miRNA contrasting folic acid and persistent organic pollutants (POPs) [8]. Interestingly, miRNA was measured in sperm cells of male rats (F1-F4) whose mothers were exposed to POPs and were then subsequently supplemented with folic acid [8]. Supplementation with folic acid partially mitigated alterations in miRNA in generations F1 through F3. In contrast, maternal smoking during pregnancy and *AHRR* methylation may be counteracted by maintaining adequate levels of folic acid during pregnancy [10]. Finally, paternal exposure to rosuvastatin showed increased DNA fragmentation, androgen depletion, and cellular damage to the testicles and epididymis in offspring. Paternal co-administration of ascorbic acid showed mitigation of reproductive damage to the offspring of rosuvastatin-exposed fathers [9].

Conducting research on human exposure to toxins is complicated. If ethical concerns are addressed, however, observational studies looking at the effects of toxins on the human body can lead to more funding and motivation for public health improvements that limit said toxin exposures [96]. Even observational studies must be tightly regulated such that participants are fully informed of the toxins to which they are exposed, participants are chosen equitably, participants are exposed to the least amount of risk possible, and the benefit of the knowledge obtained by the participants as a result of the research outweighs the risk of toxin exposure [96]. The most feasible studies would consist of retrospective analysis following toxin exposure, but for prospective studies different approaches are warranted. Because of these challenges, animal models have been widely used in scientific research to gain knowledge while limiting harm to human subjects. Animal testing for this purpose may not be as reproducible across animal species or reliable in its relation to humans as previously thought. There are increasing doubts in the ability to predict effects of toxin exposure in one species by relying on the effects shown in another species [97]. Alternatives to animal testing include but are not limited to the following: in vitro tests using cell lines, tissue samples, use of alternative organisms such as bacteria, 3-dimensional modeling and bioprinting [98]. Although these methods have shown great promise, they have yet to undergo greater regulatory testing to begin to take the place of animal testing [98]. While longitudinal human trials may investigate many dietary interventions without much ethical concern, we might encourage the further development of animal testing alternatives to research the relation of dietary intervention and toxin exposure in a clinically meaningful way.

Another consideration is that the studies collected have many confounding variables such as varying species, dietary interventions, and various data collection methods, limiting the ability to draw definite conclusions. Each study also creates a different environment for its subjects, which may alter the way the subjects’ epigenome responds to the given dietary intervention. For example, when a dietary intervention includes caloric restriction [15,19,20] or vitamin D deficiency [17], this could cause a psychological stressor on the subject due to being hungry or malnourished. Psychological stressors on their own create their own effects on the epigenome [99] which are not the focus here and, therefore, function as a confounding variable. Additionally, the biggest challenge to drawing conclusions may be that while epigenetic changes are studied in multiple generations for each study, the number of generations studied varies. Trends, therefore, are being summarized in these studies, hoping to create an informed hypothesis about variables that increase duration of epigenetic change that can then be used to inspire future research needed in this area.

Additionally, in the human trials discussing toxins [5,6,10], the dose of toxin exposure was not captured and the dietary intervention (Mediterranean Diet, provitamin A, folic acid) [5,6,10] were not controlled but only measured via adherence to diet or serum levels of the nutrient. This is due to the fact that it is unethical to administer any level of exposure to these toxins; similarly, you cannot control against multiple confounding exposures which may be present. For example, every day we encounter uncontrolled toxins such as pesticides in our food, ambient particles in the air, and impurities from water sources; at the same time, some individuals combat toxin load with lifestyle effects by exercising, taking supplements, or using a sauna. The dose of these toxins may greatly vary between each individual as well as have a significantly different effect due to an individual’s genetics. As humans have a greater variability between genetic similarity than a controlled experiment with lab animals, translating these studies to humans can be complicated.

In the evaluation of the literature for this review, the goal of the research was not to determine the maximum generational duration of epigenetic change. For example, some studies had a predetermined design to measure epigenetic modifications up to F3. If epigenetic modifications were preserved in F3, the F4 generation was not evaluated to see if the modification was preserved for yet another generation [16,18,20,22]. That said, the duration of change may be underestimated. With the understanding of the epigenome being a dynamic construct that keeps track of the changes in the environment, it may be possible that a duration of three generations is the longest duration of change that could be seen. An epigenetic modification that lasts longer than this may undermine the ability of the epigenome to remain dynamic throughout changing environmental challenges [35]. More research should be done with maximum duration of change as the primary hypothesis being tested, to see which generation shows complete loss of a dietary intervention’s epigenetic change.

## Conclusion & future directions

Our review highlights the potential of the Mediterranean Diet as a foundational intervention to epigenetically protect against environmental toxins across generations. The data we reviewed suggest that this diet, rich in protein, healthy fats, and micronutrients like provitamin A, resveratrol, and folic acid, can mitigate the harmful effects of toxins such as tobacco smoke, ethanol, and morphine by modulating DNA methylation, histone acetylation, and miRNA expression. Notably, our analysis revealed that the timing of dietary interventions – whether during pre-fertilization, pregnancy, or throughout life – does not significantly alter the persistence of these epigenetic effects, which can last for two to three generations.

The implications of these findings are profound for clinical practice. They suggest the dietary recommendations could be strategically implemented during critical periods to optimize long-term health outcomes and provide transgenerational protection. To validate these observations, future research should prioritize longitudinal, multigenerational studies that measure the epigenetic impact of the Mediterranean Diet during sensitive developmental windows.

Given the variability of the Mediterranean Diet discussed above, we would specifically suggest prioritizing a diet with a fat:protein:carb ratio of 25:35:40 as the dietary modification in future studies with an emphasis on what types of fats are considered healthy fats. This would allow for a diet of increased protein and healthy fats to be studied while also attempting to standardize what the dietary recommendation might be by setting an exact ratio. While there are specific foods that make up the Mediterranean Diet, we would first suggest a study that focuses on the ratio of the Mediterranean Diet rather than the food types in order to incorporate a large variety of cultural foods and encourage a more diverse group of participants. We would further suggest that this study look at the epigenetic modifications of AHRR methylation and the levels of systemic inflammation by measuring inflammatory markers such as CRP. Looking at AHRR methylation could show a relationship between this dietary ratio and an increased ability to clear harmful inflammatory markers from the body, including those introduced by smoking tobacco. Looking at CRP, then, is a very common measurement of overall systemic inflammation in general medical practice [100] Appropriate controls for this experiment would consist of individuals in the same household not adhering to this diet regimen.

Additionally, exploration into the cumulative effects of dietary interventions across generations and their interaction with specific environmental toxins will be crucial to refining our understanding of dietary epigenetics in preventive medicine. There may be ways to incorporate tobacco smoke exposure into this study by enrolling smokers and non smokers into the study. This would allow the study to look at tobacco smoke’s effects on systemic inflammation and AHRR methylation as well, as we saw in previous studies discussed in this review. Overall, longitudinal studies such as this will be logistically challenging to coordinate; however, if diets similar to the Mediterranean Diet ratio presented above are universally recommended in primary care settings, there may be more and more opportunity to follow patients who are already undergoing this dietary intervention and follow their families across generations.

Given the extensive protective and preventive effects of diet, there is an ongoing call to action for physicians to receive formal nutrition training or for healthcare institutions to increase access to nutritionists. This will help enhance both the quality and longevity of life for patients and their future offspring.

## Data Availability

The authors confirm that the data supporting the findings of this study are available within the article [and/or] its supplementary materials.
